# Exploring Gender Differences in Internet Addiction and Psychological Factors: A Study in a Spanish Sample

**DOI:** 10.3390/brainsci14101037

**Published:** 2024-10-19

**Authors:** Manuel Varchetta, Ginevra Tagliaferri, Emanuela Mari, Alessandro Quaglieri, Clarissa Cricenti, Anna Maria Giannini, Manuel Martí-Vilar

**Affiliations:** 1 Department de Psicologia Bàsica, Faculty of Psychology and Speech Therapy, Universitat de València, Av. Blasco Ibañez, 21, 46010 Valencia, Spain; 2 Department of Psychology, Sapienza University of Rome, Via dei Marsi 78, 00185 Rome, Italy; ginevra.tagliaferri@uniroma1.it (G.T.); e.mari@uniroma1.it (E.M.); alessandro.quaglieri@uniroma1.it (A.Q.); clarissa.cricenti@uniroma1.it (C.C.); annamaria.giannini@uniroma1.it (A.M.G.); 3 Department of Psychology and Human Sciences, Universitas Mercatorum, Piazza Mattei 10, 00186 Rome, Italy

**Keywords:** gender differences, problematic use of Internet, social media use, Internet gaming disorder, FoMO, phubbing, psychological correlates

## Abstract

Background/Objectives: Internet addiction (IA) and related behaviors, such as Internet Gaming Disorder (IGD) and social media addiction (SMA), have gained increasing research attention. Studies show gender differences, with males more likely to develop gaming-related addictions and females more prone to social media and phubbing behaviors. This study aimed to explore gender differences in Internet addiction and related behaviors in a Spanish sample, with the goal of identifying predictors and gender-specific patterns of IA. Methods: We conducted a cross-sectional study with 585 participants (265 male, 320 female) aged 18 to 35 years (M = 22.11, SD = 3.08). Data were collected using standardized questionnaires to assess IA, IGD, SMA, phubbing, Fear of Missing Out (FoMO), emotional dysregulation, personality traits, and prosociality. Correlation and regression analyses were used to identify gender-specific predictors of IA. Results: Males exhibited significantly higher scores for IA and IGD, while females showed higher scores for SMA and the “phone obsession” dimension of phubbing. No significant gender differences were found in the “communication disturbance” dimension of phubbing or in FoMO. Correlation analyses revealed significant associations between IA and psychological as well as technological variables. Gender-specific predictors of IA included social media engagement and emotional regulation for females, while gaming behaviors and communication patterns were more relevant for males. Conclusions: These findings highlight gender differences in IA, suggesting that tailored interventions should address unique online behaviors and emotional regulation challenges in males and females. Future research should refine gender-specific patterns to develop more effective, targeted prevention and treatment strategies.

## 1. Introduction

Behavioral addictions, also known as non-substance addictions or non-chemical behavioral addictions, are disorders characterized by an inability to control specific behaviors, often resulting in harmful consequences for the individual’s mental, emotional, physical, or social health [1]. Technological addictions can be found within this category. Technological addictions are a group of non-chemical behavioral addictions that involve human–machine interaction. These interactions can be either passive, such as watching television, streaming videos, or endlessly scrolling through social media, or active, such as playing computer games, shopping online, or participating in interactive chatrooms. Such behaviors often feature inducing and reinforcing mechanisms that contribute to addictive tendencies [2]. Examples of technological addictions include gambling (e.g., slot machine addiction) and Internet addiction (IA). Like other forms of addiction, technological addictions involve key components such as craving, tolerance, withdrawal, salience, mood modification, conflict, and relapse. Additionally, they are characterized by reinforcement and induction processes that promote continuous engagement with the technology [3,4].

IA refers to excessive and dysfunctional use of the Internet that interferes with daily life, mental well-being, and interpersonal relationships. According to Young [5], IA encompasses five subtypes of behaviors: compulsive online gambling, cybersex addiction, information overload addiction, Internet Gaming Disorder (IGD), and cyber-relationship addiction. In addition to Young’s classification [5], further technological addictions have been identified over time, including social media addiction (SMA) [6]. IGD is the only technological addiction that has been included in the latest version of the International Classification of Diseases (ICD-11) under the “disorders due to addictive behaviors” section [7] and proposed as a condition to be included in the “conditions for future research” section of the DSM-5 TR [8]. IA, particularly IGD and SMA, as behavioral addictions involving a lack of impulse control, have emerged as being correlated with different personality characteristics, including impulsivity, difficulty in emotional regulation, reduced social skills, and social withdrawal [9,10].

From the analysis of these addictions, two phenomena associated with them have emerged and have become quite well known in the context of Internet use [11,12]: phubbing and Fear of Missing Out (FoMO), which seem to play a role in the development and maintenance of addiction. These phenomena will be described in the following paragraphs.

Additionally, we will review the existing literature on gender differences related to Internet addiction and these associated behaviors.

### 1.1. Gender Differences in Internet Addiction

The phenomenon of IA (here defined as excessive Internet use and Internet-related addictive behaviors) has been discussed for its conceptualization and definition and is considered to lead to impairment of an individual’s psychological state (both mental and emotional), as well as the person’s scholastic, work-related, and social interactions [13]. It has been seen that gender is a controversial and much-discussed variable related to IA; for instance, when referring to a generalized form of IA, research indicates that males are more vulnerable than females [14,15,16], whereas, when analyzing specific forms (problematic use of social media or problematic smartphone use), the opposite is true [17,18,19,20]. This could be related to the different activities that can be performed online and the different preferences between males and females: while males engage in gaming, pornography sites, and file downloading [21], females are more involved in social and communication activities [22]. However, some studies have failed to reveal any significant gender differences in symptoms and severity of IA [23,24]. Overall, this suggests that gender differences in IA may be context-dependent, varying based on the specific applications or platforms being used, rather than reflecting consistent patterns across all forms of Internet use.

### 1.2. Gender Differences in Internet Gaming Disorder (IGD) and Social Media Addiction (SMA)

SMA is defined as “the inability to regulate the use of social networks, which causes negative effects on a personal and interpersonal level” [6,25], while IGD is characterized by a pattern of persistent or recurrent gaming behavior (e.g., “digital gaming” or “video gaming”) and is associated with negative consequences (e.g., social, occupational, familial, or educational) and functional impairment [7], as recognized by the World Health Organization [26].

Research shows that females spend more time on social media than males, indicating SMA, with exposure to the social world at an early age compared to males [27,28,29,30]. Females have a greater interest in using social media as tools for social interaction [28,31,32,33], while studies demonstrate that males are more vulnerable to developing IGD symptoms, especially in relation to high stress, impulsivity, hyperactivity, and poor academic performance [16,34,35]. However, the results regarding gender differences in Internet-related behaviors are not always consistent, and there is a need for further studies to better understand these complexities.

### 1.3. Gender Differences in Fear of Missing Out (FoMO)

FoMO is defined as “a pervasive apprehension that others might be having rewarding experiences from which one is absent” [36]. FoMO can be conceptualized as a negative emotional state resulting from the individual’s unmet relational needs [36]; hence, individuals with satisfied relational needs should experience lower levels of FoMO. Regarding gender differences, females report greater relational satisfaction and engagement in maintaining relationships through social media; therefore, they should experience less FoMO. However, Elhai et al. [37] demonstrated that FoMO was more strongly correlated with the female gender, in line with most research in the literature [37,38,39]. Conversely, Przybylski et al. [36] highlighted higher FoMO scores in males with low life satisfaction levels, who exhibited excessive use of social media. Nevertheless, other studies [40,41,42] have not consistently demonstrated significant gender differences in FoMO, highlighting the need for further research to clarify these findings.

### 1.4. Gender Differences in Phubbing

The term “phubbing” is a blend of the words “phone” and “snubbing”, and it refers to the phenomenon of snubbing an interlocutor in the context of social contact by focusing one’s attention on the phone rather than the person one is conversing with [12]. Phubbing positively correlates with loneliness, understood as a negative feeling of social exclusion [43]; indeed, directing one’s attention toward the phone increases the sense of exclusion from the social environment [44]. Regarding gender differences, several studies [12,43,45] demonstrate that gender can moderate the relationships between phubbing behavior and smartphone addiction (e.g., social media, gaming, and Internet use). Specifically, in the female population, phubbing correlates with smartphone addiction and SMA, while in the male population, it is more associated with IA and IGD [46]. Overall, females score higher than males in phubbing behavior, indicating that the female gender uses smartphones more as facilitators of social interactions, while for the male gender, they serve a more instrumental function [45]. However, the inconsistencies in findings across studies highlight the need for further research to better understand the dynamics of phubbing behavior and its implications across genders.

### 1.5. Emotional (Dys)regulation and Internet Addiction

Emotional regulation is defined as a set of skills that include awareness, understanding, and acceptance of emotions; the ability to manage impulsive behaviors; and the ability to adopt valid regulation strategies to achieve personal goals and adapt to situations [47]. Some studies [48,49] have highlighted how excessive Internet use is used as a coping strategy to compensate for or alleviate negative emotions. Those who have difficulty regulating emotions seem to be more vulnerable to developing problematic/excessive Internet use because the Internet becomes a means—especially for younger individuals—to regulate emotions [50,51,52,53].

A strong association between emotional dysregulation and excessive Internet use has emerged in the literature [54,55,56], indicating that individuals addicted to the Internet report greater difficulties in understanding and describing their own and others’ emotions and controlling their impulsive behaviors in response to negative affective states [19]. Regarding emotional regulation, males exhibit higher levels of emotional dysregulation than females, who demonstrate greater abilities in understanding, awareness, and emotional expression [57,58].

### 1.6. Prosociality and Internet Addiction

Prosocial behavior can be defined as voluntary behavior aimed at benefiting another individual or group [59]; it appears in preschool-age children (two years) and increases in frequency and variety throughout life [60]. Prosocial behavior is based on the concept of helping, sharing, and comforting others and is influenced by both situational and individual factors [61]; therefore, prosociality contributes to reducing the likelihood of engaging in antisocial behaviors [62]. Due to its communal and social nature, engaging in prosocial behaviors could serve as a protective factor against the development of behavioral addictions, as the latter are more related to an individual sphere, where the subject is constantly focused on performing addiction-related behaviors compared to the community context [63]. For this reason, prosocial behaviors could reduce the risk of developing IA [64].

Regarding gender differences, the literature highlights that females engage more in prosocial behaviors than males [65]. This seems to derive from the nature of female behavior, which is more oriented towards nurturing compared to the male group, which is more justice-oriented [66]. Gender stereotypes play a fundamental role in this context, as they influence socialization during growth [67]. Females are more socialized to show nurturing and caring behaviors, while males are socialized with the pressure to inhibit this prosocial behavior [68,69].

### 1.7. Impulsivity and Internet Addiction

Impulsivity is described as a tendency to respond in an unplanned manner to both internal and external stimuli, without considering the possible negative consequences of one’s actions [70]. Impulsivity is a core feature observed in various psychiatric disorders, where individuals struggle to regulate their actions, often acting hastily without considering the potential negative consequences [71,72]. This trait is commonly associated with conditions such as personality disorders, eating disorders [73] substance abuse [74], and, not least, IA [75]. A strong association between impulsivity and IA has emerged in the literature [48,76]; indeed, for those who have difficulty inhibiting behaviors, the Internet becomes the place to constantly receive rewards, gratification, and immediate reinforcements [77]. Individuals with IA exhibit high impulsivity and the presence of other comorbid disorders [78]. Additionally, Lee et al. [79] found that impulsivity can be a risk factor for IA and pathological gambling. Gender differences in impulsivity are well established, with males displaying higher levels of impulsive behavior, particularly in sensation-seeking and risk-taking contexts [80]. Although there are some significant findings regarding the relationship between gender differences in impulsivity and IA, this area warrants further investigation to fully understand the nuances of how these factors interact.

### 1.8. Main Hypotheses

Despite increasing recognition, gaps remain in understanding the gender-specific mechanisms that contribute to these addictions and how factors such as emotional regulation, personality traits, and impulsivity interact with IA. Furthermore, although the literature highlights notable differences in SMA [27,28,29,30,31], IGD [34,35], FoMO [37,38,39], and phubbing [45], conflicting results and inconsistencies persist.

Building upon the prior study conducted by Mari et al. [81] that investigated gender differences in Internet addiction within an Italian sample, this research aims to extend the inquiry by examining similar relationships in a Spanish sample and address some of the existing gaps in the literature. This study seeks to provide additional insights into the associations between gender and Internet addictive behaviors, specifically focusing on unraveling potential differences related to psychological factors such as impulsivity, prosocial behaviors, and emotional regulation and recognition. That is precisely why we aim to replicate the hypotheses of the previous study, reinforcing the robustness of its findings. By applying similar hypotheses to a Spanish sample, we strive to validate and build upon the existing knowledge regarding gender differences in IA, SMA, FoMO, phubbing, and various psychological variables. This research adds depth to our understanding of these relationships, contributing to the broader context of cross-cultural research in this domain.

**Hypothesis** **1 (H1):** *There will be significant differences between males and females in various aspects of Internet-related behaviors and psychological variables. Specifically, the following differences are expected: *

*a.*  *Males are anticipated to exhibit higher levels of IA;*
*b.*  *Males are expected to display higher levels of IGD, while females are predicted to have higher levels of SMA;*
*c.*  *Females are expected to demonstrate higher levels of FoMO;*
*d.*  *Females are hypothesized to exhibit a greater tendency to experience phubbing;*
*e.*  *Females are anticipated to demonstrate higher levels of prosociality.*

**Hypothesis** **2 (H2):** *We anticipate significant correlations between Internet addiction and the examined variables, including SMA, IGD, FoMO, phubbing, emotional regulation, and personality traits, with variations based on gender.*

**Hypothesis** **3 (H3):** *The variables under investigation, including SMA, IGD, FoMO, phubbing, emotional regulation, and personality traits, are expected to serve as predictors of IA within distinct male and female groups.*

## 2. Materials and Methods

### 2.1. Participants

Initially, the study included 596 participants recruited through a non-probabilistic convenience sampling method. To qualify for inclusion, participants had to be at least 18 years old and residing in Spain. Out of the initial participants, eleven did not complete the full questionnaire, leading to the decision to include only fully completed responses in the analysis to maintain data integrity and reliability. All participants provided informed consent. Consequently, the final sample consisted of 585 participants (265 males and 320 females), aged between 18 and 35 years (M = 22.11, SD = 3.08). Males accounted for 45.3% of the sample (M = 22.76, SD = 3.65), and females made up 54.7% (M = 21.81, SD = 2.93), which falls within the acceptable range of 40–60% to minimize gender bias. Gender was measured as a categorical variable, and while participants were given the option to indicate other gender identities, all respondents selected the binary options.

### 2.2. Procedure

Participants were recruited online and voluntarily responded to the survey anonymously. They accessed the survey through a designated link on the Qualtrics Online Platform. The recruitment was incidental, meaning that the participants were those who had access to the survey link and chose to participate. This may explain the high proportion (85%) of students in the sample. No incentives were offered for participation. To minimize bias, informed consent was obtained before the survey began, emphasizing that responses were anonymous and that participants could leave the survey at any point without consequence. This anonymity was aimed at reducing socially desirable responding. Additionally, participants completed the survey in various environments (e.g., home, university, or work) using devices of their choice (PCs, smartphones, or tablets). The average response time for completing the survey was approximately 20 min.

Expedited ethics approval was obtained from the Institutional Board of the Comité de Ética of the University of Valencia (IRB 15910/2021) in accordance with the principles of the Declaration of Helsinki.

### 2.3. Measures

#### 2.3.1. Internet Addiction Test (IAT)

The Internet Addiction Test (IAT), developed by Young [48], is a widely used instrument designed to measure addictive Internet use. Comprising 20 items based on the DSM-IV criteria, it assesses aspects such as the fear of life without the Internet and attempts to cut down online time. Responses are recorded on a five-point Likert scale ranging from 1 (very rarely) to 5 (very frequently). Scores are categorized as follows: (1) Normal users or users without problems (<40 points) and (2) problematic Internet users (≥40 points) [82]. The Spanish version by Fernández-Villa et al. [83] was used in this study, with a Cronbach’s alpha value of 0.86.

#### 2.3.2. Social Media Engagement Scale (SMES)

The Social Media Engagement Scale (SMES), developed by Przybylski et al. [36], is designed to measure the frequency of social network use during daily activities. Respondents rate their engagement using an eight-point Likert-type scale, ranging from 1 (no day last week) to 8 (every day last week). To compute individual scores, responses are averaged, resulting in a mean score for each participant. The mean scores indicate the average frequency of social media use during these daily activities. The scale has demonstrated strong reliability, with Cronbach’s alpha coefficients ranging 0.79.

#### 2.3.3. Bergen Social Media Addiction Scale (BSMAS)

The Bergen Social Media Addiction Scale (BSMAS), developed by Andreassen et al. [84] and based on Griffiths’ [85] six dimensions of addiction, comprises six items evaluating various aspects of social media addiction such as salience, mood modification, tolerance, withdrawal, conflict, and relapse. Sample items include queries such as “How often during the last year have you used social media so much that it has had a negative impact on your job/studies?” and “How often during the last year have you felt an urge to use social media more and more”? Participants rate their responses on a 5-point Likert scale, with higher scores indicating more severe symptoms of social media addiction. A total score of 24 or above suggests a potential clinical diagnosis of SMA. The Spanish version of the BSMAS, adapted by Vallejos-Flores et al. [41], was utilized in the current study. This version has demonstrated good internal consistency, with Cronbach’s alpha coefficient reported at 0.87, indicating reliable measurement of social media addiction symptoms within Spanish-speaking populations [86].

#### 2.3.4. Internet Gaming Disorder Scale—Short Form (IGDS9-SF)

The Internet Gaming Disorder Scale-Short Form (IGDS9-SF), developed by Pontes and Griffiths [87], is designed to evaluate the severity of IGD and its associated consequences by assessing both online and offline gaming activities spanning a 12-month period. The scale comprises nine items, each corresponding to the nine core criteria outlined in the DSM-5 [88] for diagnosing IGD. Participants provide responses on a five-point Likert scale, ranging from 1 (never) to 5 (very often), to indicate the frequency of their gaming-related behaviors. A cutoff score of 21 is applied, with higher scores on the scale denote a greater degree of gaming disorder symptomatology. For this study, the Spanish version of the IGDS9-SF, validated by Beranuy et al. [89], was utilized. The scale demonstrated good internal consistency, with a reported alpha reliability coefficient of 0.72, indicating its reliability in assessing IGD symptoms within Spanish-speaking populations.

#### 2.3.5. Fear of Missing Out Scale (FoMOs)

The Fear of Missing Out Scale (FoMOs), developed by Przybylski et al. [36], is a self-report questionnaire comprising 10 items aimed at assessing individuals’ experiences of a pervasive apprehension regarding others’ engagement in rewarding activities and positive relationships in their absence. Sample items include statements such as “I get worried when I find out my friends are having fun without me”. Respondents indicate their level of agreement on a 5-point Likert scale, ranging from 1 (not true of me) to 5 (extremely true of me). Higher average scores on the FoMOs, on a scale from 1 to 5, signify higher levels of Fear of Missing Out. In this study, the Spanish version of the FoMOs, as developed by Gil et al. [40], was utilized. The scale demonstrated good internal consistency, with a Cronbach’s alpha coefficient at 0.80.

#### 2.3.6. Phubbing Scale

The Phubbing Scale (PHUB), developed by Karadag et al. [43], comprises ten items aimed at assessing phubbing behavior. Participants rate their responses on a five-point Likert scale, ranging from 1 (never) to 5 (always). The scale evaluates two primary factors: “communication disturbance” and “phone obsession”. “Communication disturbance” items reflect behaviors such as “My eyes start wandering on my phone when I’m together with others”, while “phone obsession” items gauge behaviors such as “When I wake up in the morning, I first check the messages on my phone”. Higher means scores (cutoff = 2.5) reflect a greater tendency toward phubbing behaviour. In this study, the Spanish version of the PHUB, validated by Blanca and Bendayan [90], was employed. The reliability of the scale was assessed using Cronbach’s alpha coefficient, which indicates internal consistency. For the “communication disturbance” factor, the Cronbach’s alpha coefficient was 0.87. Similarly, for the “phone obsession” dimension, the Cronbach’s alpha coefficient was 0.86, suggesting acceptable internal consistency for this factor as well.

#### 2.3.7. Difficulties in Emotion Regulation Scale (DERS)

The Difficulties in Emotion Regulation Scale (DERS), developed by Gratz and Roemer [47], is a self-report questionnaire designed to evaluate various aspects of difficulties in regulating emotions. It consists of 36 items that assess six dimensions of emotion regulation: non-acceptance, goal-directed behavior, impulse control, limited access to effective emotional regulation strategies, lack of emotional awareness, and lack of emotional clarity. In this study, the Chilean short version of the DERS, proposed by Guzmán González et al. [91] was utilized. This version comprises 25 items and provides a global score, calculated as the average of all item scores, as well as scores for each scale representing different dimensions of emotional regulation (non-acceptance, goal-directed behavior, impulse control, limited access to effective emotional regulation strategies, lack of emotional awareness, and lack of emotional clarity). Participants rated their responses on a Likert scale ranging from 1 to 5, where higher scores indicate greater difficulties in emotional regulation. The total Cronbach’s alpha coefficient for the Spanish sample was reported at 0.83, indicating high internal consistency reliability for the scale. Regarding the factors of non-acceptance, goal-directed behavior, impulse control, limited access to effective emotional regulation strategies, lack of emotional awareness, and lack of emotional clarity, the Cronbach’s alpha coefficients were 0.91, 0.89, 0.91, 0.87, 0.86, and 0.82, respectively.

#### 2.3.8. Big Five Inventory (BFI)

The Big Five Inventory-15 (BFI-15), originally developed by Gerlitz and Schupp [92], is a self-report questionnaire aimed at assessing five fundamental personality dimensions: extraversion, agreeableness, conscientiousness, neuroticism, and openness to experience. Participants, consisting of Peruvian university students across three Spanish-speaking samples, provided responses to statements such as “I see myself as a person that is reserved” and “I see myself as a person that tends to find fault with others” using a 5-point Likert scale, ranging from 1 (strongly disagree) to 5 (strongly agree). Mean scores were calculated for each personality dimension. Dominguez-Lara and Merino-Soto [93] adapted the scale for the Spanish-speaking context. The reliability of this specific scale, as assessed by Cronbach’s alpha, was 0.93. The Cronbach’s alpha coefficients for the specific dimensions of the BFI in this study were as follows: extraversion: 0.87; agreeableness: 0.88; conscientiousness: 0.84; neuroticism: 0.83; openness to experience: 0.89 These values indicate strong internal consistency for each dimension measured.

#### 2.3.9. Prosociality Scale (PS)

The Prosociality Scale (PS), created by Caprara et al. [94], is a 16-item questionnaire utilized in the current study. It evaluates various behaviors and feelings related to four types of prosocial actions: sharing, helping, taking care of others, and feeling empathic towards their needs. Participants rate each prosocial behavior item on a five-point Likert scale, ranging from 1 (never/almost never true) to 5 (almost always/always true). Higher scores indicate higher levels of prosociality. This study employed the adapted version of the scale, developed by Rodriguez et al. [95]. This adaptation ensures linguistic and cultural appropriateness for Spanish-speaking participants, thereby enhancing the validity and reliability of the instrument within this specific cultural and linguistic context. The Cronbach’s alpha coefficient for this adapted version was reported as 0.71, indicating good internal consistency reliability for measuring prosociality among Spanish-speaking individuals in the study.

#### 2.3.10. UPPS-P Impulsivity Behavior Scale (UPPS)

The UPPS-P Impulsivity Behavior Scale (UPPS) utilized in this study is grounded in the work of Whiteside and Lynam [96], assessing five dimensions of impulsivity: positive urgency, negative urgency, lack of perseverance, lack of premeditation, and sensation seeking. The short Spanish version of the UPPS Scale, adapted from Candido et al. [97], consists of 20 items evaluating five impulsivity traits, each represented by four items. These traits encompass negative urgency (items 4, 7, 12, and 17), lack of premeditation (items 1, 6, 13, and 19), lack of perseverance (items 5, 8, 11, and 16), sensation seeking (items 3, 9, 14, and 18), and positive urgency (items 2, 10, 15, and 20). Mean scores are calculated for each dimension. The Cronbach’s alpha total score was 0.90. The Cronbach’s alpha coefficients for the dimensions assessed in the study are as follows: negative urgency: 0.84; positive urgency: 0.79; lack of premeditation: 0.81; lack of perseverance: 0.90; sensation seeking: 0.83. These values indicate good to excellent internal consistency for each dimension measured in the study.

### 2.4. Data Analysis

The study utilized the Statistical Package for Social Science (SPSS; version 26.0 IBM SPSS, Armonk, NY, USA) for conducting statistical analyses. Descriptive statistics were computed for various demographic variables including gender, age, education, marital status, profession, and family type, presenting key metrics such as mean, median, and standard deviation.

To compare questionnaire scores between distinct groups, a Multivariate Analysis of Variance (MANOVA) was performed with problematic Internet use, emotion, and self-reported personality measures as dependent variables and gender as a fixed factor. Bonferroni corrections were applied for multiple comparisons between genders. The significance level for all tests was set at *p* < 0.05.

Effect sizes were calculated using eta squared (η^2^), with values interpreted according to Cohen’s [98] guidelines: η^2^ values of 0.01, 0.06, and 0.14 represent small, medium, and large effects, respectively.

Parametric assumptions were checked using variance ratio tests and Levene’s tests for equality of variances. For each gender separately, partial Pearson’s correlations were conducted, and the significance of the correlation coefficients between the two gender groups was compared using Fisher’s z test. Subsequently, stepwise linear regression analyses were performed to investigate relationships between variables and Internet addiction. Multicollinearity was assessed using the variance inflation factor (VIF) and tolerance. Statistical significance was set at *p* < 0.05, and normality of data distributions was verified prior to analyses. All statistical analyses were performed on de-identified data in accordance with ethical standards.

## 3. Results

### 3.1. Descriptive Statistics and Mean Differences Between Variables in the Different Groups

The study conducted an analysis comparing mean scores between male and female groups for each variable under consideration.

Males showed significantly higher mean scores than females on the IAT (F_(1,582)_ = 5.15, *p* = 0.006, η^2^ = 0.02) and IGDS (F_(1,582)_ = 30.27, *p* < 0.001, η^2^ = 0.09). Conversely, females had significantly higher scores on the BSMAS than males (F_(1,582)_ = 8.61, *p* < 0.001, η^2^ = 0.03), the SMES (F_(1,582)_ = 6.54, *p* = 0.002, η^2^ = 0.02), and the “phone obsession” dimension of phubbing (F_(1,582)_ = 8.91, *p* < 0.001, η^2^ = 0.03).

No statistically significant differences were observed between the male and female groups regarding the “communication disturbance” dimension of phubbing (F_(1,582)_ = 0.47, *p* = 0.627, η^2^ = 0.00) and FoMO (F_(1,582)_ = 0.50, *p* = 0.481, η^2^ = 0.00)

All the data regarding technological variables are shown in the Table 1.

The differences in mean scores for psychological variables are presented in Table 2.

Specifically, females exhibited higher levels of DERS than males (F_(1,582)_ = 9.03, *p* < 0.001, η^2^ = 0.030). Concerning the BFI, females showed higher levels of “neuroticism” (F_(1,582)_ = 3.60, *p* < 0.05, η^2^ = 0.01), “agreeableness” (F_(1,582)_ = 3.18, *p* < 0.05, η^2^ = 0.01), and “conscientiousness” (F_(1,582)_ = 12.57, *p* < 0.001, η^2^ = 0.04). No significant differences were observed in “extraversion” (F_(1,582)_ = 0.28, *p* = 0.756, η^2^ = 0.00) and “openness to experience” (F_(1,582)_ = 2.54, *p* = 0.080, η^2^ = 0.01).

Regarding prosocial behavior, females exhibited higher scores than males (F_(1,582)_ = 40.89, *p* < 0.001, η^2^ = 0.07).

Finally, concerning the UPPS, females showed higher levels of the “negative urgency” (F_(1,582)_ = 6.19, *p* = 0.002, η^2^ = 0.02) and “positive urgency” dimensions (F_(1,582)_ = 3.61, *p* < 0.05, η^2^ = 0.01). Conversely, males exhibited higher levels of the “lack of perseverance” dimension (F_(1,582)_ = 12.63, *p* < 0.001, η^2^ = 0.04) and sensation seeking dimension (F_(1,582)_ = 6.48, *p* = 0.002, η^2^ = 0.02). No statistically significant differences were observed regarding the “lack of premeditation” dimension (F_(1,582)_ = 1.75, *p* = 0.174, η^2^ = 0.01).

### 3.2. Correlations Between Variables in the Male and Female Groups

Partial Pearson’s correlations were computed to examine the connections between the primary variables under investigation in the study. Separate correlation analyses were conducted for each group to explore potential relationships between the total score on the IAT and other variables.

Furthermore, significant correlation coefficients from the two groups were subjected to comparison using Fisher’s z test.

Concerning the technological variables, IAT scores were positively related to scores on the BSMAS, the SMES, the IGDS, the “communication disturbance” and “phone obsession” dimensions of phubbing, and FoMO. To enhance clarity, all significant correlations pertaining to both male and female groups were documented in Table 3. The only statistically significant difference between correlations for the male and female groups was found between IAT and IGDS scores, where the former showed significantly higher correlations than the latter (z = 3.882, *p* < 0.001).

In Table 4, we present all the correlations regarding IAT scores and the psychological variables included in the study. Both male and female participants presented statistically significant positive correlation between the IAT and the DERS; the “neuroticism” dimension of BFI; and the “negative urgency”, “positive urgency”, and “lack of perseverance” dimensions of the UPPS. For the male group, but not for females, we observed a weak positive correlation between IAT scores and the lack of premeditation dimension of the UPPS (r = 0.130, *p* < 0.01).

The two groups showed weak statistically significant negative correlation between IAT scores and the extraversion, agreeableness, and conscientiousness dimensions of the BFI. For the male group, but not for females, we observed a weak negative correlation between IAT scores and the openness to experience dimension of the BFI (r = −0.094, *p* < 0.01).

In neither of the groups, statistically significant correlations were detected between IAT scores and prosociality, nor between IAT scores and the sensation seeking dimension of the UPPS. No statistically significant difference was found in these correlations.

### 3.3. Linear Regression Analyses

In the analysis focused on the male group (as shown in Table 5), stepwise multiple linear regression was employed to determine which variables could effectively forecast the emergence of symptoms associated with Internet addiction. The results revealed that a set of five predictors accounted for 64.8% of the variance (R^2^ = 0.684, F_(5,182)_ = 66.98, *p* < 0.001).

Specifically, the study found that the Behavioral Social Media Addiction Scale (BSMAS) emerged as a significant predictor of Internet Addiction Test (IAT) scores (β = 0.436, *p* < 0.001). Furthermore, Internet Gaming Disorder (β = 0.319, *p* < 0.001), “phone obsession” associated with phubbing (β = 0.164, *p* < 0.005), and the awareness (β = 0.177, *p* < 0.005) and non-acceptance (β = −0.124, *p* < 0.05) dimensions of the DERS also exhibited significant predictive relationships with IAT scores.

In the analysis specific to the female group (referenced in Table 6), the findings revealed that four predictors collectively accounted for 58.7% of the variance (R^2^ = 0.587, F_(4,120)_ = 42.616, *p* < 0.001). Notably, there were no significant concerns regarding multicollinearity issues within the regression model.

The study highlighted that the BSMAS emerged as a notable predictor of IAT scores (β = 0.593, *p* < 0.001). Additionally, “communication disturbance” linked with phubbing (β = 0.255, *p* < 0.001), the IGDS (β = 0.182, *p* < 0.01), and prosociality (β = −0.156, *p* < 0.05) exhibited significant associations with IAT scores among the female group.

## 4. Discussion

Regarding the descriptive statistics of this study, the average scores of the variables analyzed were noteworthy. The IA and SMA scores were just below the threshold for being considered alarming behaviors. This indicates that both males and females are experiencing elevated levels of engagement with online platforms, which may require further attention. Indeed, the level of engagement with social media appears to be consistently high, indicating that for the majority of participants the use of social media remains steady even during key daily activities. This suggests that social media are not just a form of entertainment but rather a pervasive element in everyday life. Additionally, the prevalence of phubbing—where individuals use their phones in social settings—appears to be a common behavior across both participant groups, suggesting a widespread normalization of this conduct.

When examining psychological variables, the elevated mean scores for neuroticism and impulsivity stand out as potential risk factors for the development of dependency-related behaviors. These traits may predispose individuals to struggle with emotional regulation and impulsive decision-making, further contributing to the risk of technological addiction [48,76,81].

On the other hand, it is essential to highlight the relatively high levels of agreeableness, prosociality, and conscientiousness within the sample. These traits are often viewed as protective factors or resources that can be leveraged in interventions aimed at preventing or treating potential addictive behaviors [27,64]. Individuals exhibiting higher levels of these qualities may be better equipped to seek social support and engage in healthier coping strategies.

However, the dispersion of scores, which is indicated by the standard deviation, is critical for understanding variability within each group. A higher standard deviation reflects a broader range of responses, implying an important variability between participants’ behavior. This draws the attention to the importance of considering not only the average trends but also the individual differences that characterize this convenience sample.

Building upon the study conducted by Mari et al. [81] investigating gender differences in Internet addiction within an Italian sample, our research aimed to extend the inquiry by examining similar relationships in a Spanish sample. By taking as a starting point the hypotheses of the previous study, we aimed to strengthen its findings by helping to broaden the research in this field.

Our findings supported the first hypothesis, revealing significant differences between males and females in various aspects of Internet-related behaviors and psychological variables. Consistent with our reference study, males exhibited higher mean scores for IA with a small effect size and IGD with a medium effect size.

This aligns with existing literature indicating that males tend to engage more in online gaming and other Internet-related activities than females [99,100,101].

These findings suggest that while the differences between males and females are statistically significant, the practical significance of these differences, particularly in IA, may be limited. Instead, the differences observed between genders for IGD seems to be more substantial. Conversely, although with a limited effect size, females demonstrated higher mean scores in SMA, SMES scores, and the “phone obsession” dimension of phubbing. These findings highlight a stronger inclination towards social media usage and dysfunctional use of the phone among female participants [84,102], suggesting that gender influences Internet-related behaviors, with males favoring gaming while females engage more with social media. This may reflect societal norms, where males are encouraged to pursue competitive online activities and females focus on interpersonal connections. However, the practical significance of these differences is modest, indicating that these behaviors, though more pronounced in females, may not differ drastically across genders.

It is noteworthy that, against our predictions, no statistically significant differences were observed between male and female groups regarding the “communication disturbance” dimension of phubbing and FoMO. The same results have been observed by Beyens et al. [38]. They found that while there were overall differences in smartphone use patterns between males and females, gender did not significantly predict communication disturbance or FoMO. Accordingly, Gil et al. [40] found that while males generally reported higher levels of IA, gender differences were not consistent across all dimensions of Internet-related behaviors. Specifically, they did not find significant gender differences in variables related to communication disturbance and FoMO. This data provides additional support for the notion that certain aspects of Internet-related behaviors may not vary significantly by gender [103], but could be influenced by factors beyond gender, including cultural or environmental contexts.

The observed differences in mean scores for psychological variables shed light on the nuanced aspects of gender differences in technological addiction and psychological correlates. Consistent with previous literature, females exhibited higher levels of emotional dysregulation, as indicated by their elevated scores on the DERS [104]. This suggests that females may experience greater challenges in regulating their emotions, which could contribute to their susceptibility to technological addiction, including social media usage [49,105]. Indeed, social media could potentially serve as an emotional regulation strategy for female participants: providing immediate yet temporary relief, these tools could allow them to cope with negative emotions or stress through constant online interactions and social validation.

Moreover, the higher levels of neuroticism, agreeableness, and conscientiousness among females, as measured by the BFI, indicate a predisposition towards emotional sensitivity, interpersonal harmony, and conscientious behavior [106]. These traits may influence females’ engagement with technology and social media platforms, potentially contributing to higher levels of SMA and smartphone addiction [102]. Another study that supports the association between personality traits and technology use among females is the research conducted by Zhou et al. [107], in their investigation of SMA among college students, they found that higher levels of neuroticism and agreeableness were significantly associated with greater susceptibility to SMA among female participants. This suggests that certain personality traits, such as neuroticism and agreeableness, may predispose females to engage more heavily with social media platforms, leading to higher levels of addiction.

In contrast, males exhibited higher levels of sensation seeking and lack of perseverance, which are associated with impulsivity and risk-taking behaviors [96]. This propensity towards sensation seeking and lack of perseverance may predispose males to engage in excessive Internet gaming and seek novel experiences online, contributing to their higher scores on measures of IA and IGD [105]. Males’ higher levels of sensation seeking, and lack of perseverance may manifest in their tendency to engage in riskier online behaviors and struggle with maintaining focus and persistence in task completion [108].

Accordingly, research by Brand et al. [109] highlights the association among impulsivity, lack of perseverance and IGD in male gamers.

The higher levels of negative urgency among females, as measured by the UPPS, suggest that females may be more prone to engage in impulsive behaviors in response to negative emotions or distress [110]. This heightened impulsivity could drive females to seek refuge in online activities as a coping mechanism, potentially exacerbating their risk of technological addiction [111]. Several studies have demonstrated the existence of the relationship between negative urgency and IA, particularly among females [102,112]. Overall, the results indicated significant gender differences in several measures, including the IAT, DERS, and UPPS. Although, the gender effect on these variables could be limited, as indicated by the small effect size, it is important to consider their practical significance, particularly in designing interventions. For example, males demonstrated higher scores on the IGDS and some dimensions of the UPPS, which suggests that interventions aimed at this group may benefit from focusing on impulse control and managing gaming-related behaviors.

Conversely, females scored higher on the DERS and BSMAS, indicating that interventions for this group should focus more on emotional regulation strategies and managing social media use, as difficulties in emotional management could exacerbate maladaptive behaviors in online environments. Understanding these gender-specific patterns provides valuable insights for designing tailored intervention programs that address the distinct needs of different populations, ultimately improving their effectiveness in reducing problematic Internet use.

Hypothesis 2 was supported by our findings, The examination of correlations between primary variables in our study provides valuable insights into the intricate relationships among IA, technological variables, and psychological factors.

Our analyses revealed several significant correlations between the total score of the IAT and various technological variables. Specifically, the IAT exhibited positive relationships with the SMA, the SMES, the IGD, the “communication disturbance” and “phone obsession” dimensions of phubbing, and FoMO. These findings have been observed in the study of Mari et al. [81] considering an Italian sample and corroborate previous research indicating the multifaceted nature of Internet addiction and its links to diverse online behaviors [87,113].

As already observed in Mari et al. [81], of particular note was the statistically significant difference in correlations between IAT and IGDS scores, where males exhibited higher correlations compared to females. This suggests a potentially stronger association between Internet addiction and online gaming behaviors among males, underscoring gender-specific patterns in technological addiction [103].

Further analyses explored the correlations between IAT scores and psychological variables. Both male and female participants demonstrated positive correlations between IAT scores and DERS sores; the “neuroticism” dimension of the BFI; and the “negative urgency”, “positive urgency”, and “lack of perseverance” dimensions of the UPPS. These findings align with previous studies highlighting the role of emotional dysregulation and impulsivity in IA [81,109,111,114].

Interestingly, while both groups exhibited weak negative correlations between IAT and certain dimensions of the BFI (extraversion, agreeableness, conscientiousness), only the male group displayed a negative correlation with the “openness to experience” dimension. This gender-specific pattern suggests differential personality traits influencing Internet-related behaviors, warranting further exploration [106].

Notably, no significant correlations were observed between IAT scores and prosociality, nor between IAT scores and the sensation seeking dimension of the UPPS. One potential explanation is that the measures used to assess Internet addiction may not capture all dimensions of online behavior or its relationship with social behavior and sensation-seeking tendencies accurately. IA often involves various components, including excessive gaming, social media use, online shopping, and information seeking, among others. Prosocial behavior and sensation seeking may not directly align with these specific facets of Internet addiction measured by standard scales such as the IAT.

Furthermore, individual differences in how people engage with the Internet may play a role. For example, some individuals may primarily use the Internet for social interaction and networking, while others may use it for entertainment, information, or professional purposes. These differences in Internet usage patterns can influence the extent to which Internet addiction relates to prosociality or sensation-seeking tendencies. These findings imply that Internet addiction may not directly relate to all aspects of social behavior and sensation-seeking tendencies, highlighting the need for nuanced understanding in conceptualizing technological addiction [96].

Our study provided evidence in support of Hypothesis 3, indicating that the variables under investigation served as predictors of IA within distinct male and female groups. The examination of predictors for symptoms associated with IA sheds light on the intricate dynamics of online behaviors, particularly among distinct gender groups.

In the context of the male group analysis, a combination of five predictors significantly contributed to 64.8% of the variance in IAT scores. As already observed in Mari et al. [81] and in further previous research [27,115,116,117,118], SMA and IGD emerged as a predictor, underscoring the role of social media and gaming online engagement in male participants’ susceptibility to Internet addiction [103]. Furthermore, “phone obsession” associated with phubbing exhibited significant predictive relationships with IAT scores.

This indicates that excessive smartphone usage contributes substantially to problematic Internet behaviors among males [102,111].

Moreover, our study underscores the importance of emotional regulation in understanding male IA. The dimensions of “awareness” and “non-acceptance” within the DERS emerged as significant predictors of IAT scores among male participants. This suggests that males who experience challenges in recognizing and accepting their emotions are more susceptible to developing problematic Internet use patterns. These findings resonate with existing literature emphasizing the intricate interplay between emotional regulation difficulties and addictive behaviors across various domains, including Internet use [49,108].

Similarly, in the female group analysis, four predictors collectively accounted for 58.7% of the variance in IAT scores. Notably, the BSMAS emerged as a significant predictor, reflecting the pivotal role of social media engagement in female participants’ susceptibility to IA [49,84]. Furthermore, “communication disturbance” associated with phubbing, IGD, and prosociality exhibited significant associations with IAT scores among females. Interestingly, the negative association between prosociality and IAT scores may suggest that females who engage in more prosocial behaviors may be less prone to problematic Internet use [111]. These results are slightly different from the data obtained in the Italian sample [81] which found BSMAS, communication disturbance and lack of perseverance dimension as predictors of IA.

However, these findings align with previous research indicating the prominent role of social media engagement, [84] online gaming behaviors [114,119], and communication patterns [120] in driving Internet addiction across gender groups.

These outcomes are important for several reasons. First, they provide valuable insights for mental health professionals and educators, highlighting the need for gender-specific interventions that address the unique predictors of IA in males and females. For instance, programs targeting males may benefit from focusing on emotional regulation strategies and managing excessive smartphone use, while interventions for females might emphasize promoting prosocial behaviors and managing social media engagement.

Second, understanding these predictors can inform public health policies aimed at reducing Internet addiction, particularly in vulnerable populations. By recognizing the role of specific psychological and social variables, policymakers can develop targeted initiatives that address the underlying factors contributing to IA.

Finally, these findings underscore the relevance of considering gender differences in research and intervention design. As Internet usage patterns continue to evolve, it is crucial to adopt a nuanced approach that considers the distinct motivations and risks associated with Internet addiction for different genders.

## 5. Limitations and Future Directions

This study sheds light on gender differences in IA and associated psychological variables within a Spanish context. However, certain limitations must be acknowledged to contextualize our findings and to guide future research in this field.

One limitation of our study is the reliance on convenience sampling, which may introduce biases and limit the generalizability of our findings to broader populations. Future research could benefit from employing more diverse and representative samples to enhance the external validity of the results.

The cross-sectional nature of our study precludes the establishment of causal relationships between variables. Longitudinal studies would be beneficial in elucidating the temporal dynamics and causal pathways underlying Internet addiction and its correlates over time.

Additionally, the reliance on self-report measures for assessing Internet addiction and psychological variables may introduce response bias, including social desirability effects. Participants might unintentionally adjust their answers to conform to perceived social expectations or misremember certain behaviors, further complicating data accuracy. Future research could benefit from incorporating objective measures, such as tracking actual online behavior or using psychophysiological assessments and multi-method approaches to enhance the reliability and validity of the findings.

Another potential limitation of the study is that it did not consider the impact of the COVID-19 pandemic, which significantly increased the reliance on digital platforms for communication, education, and entertainment. This surge in Internet usage during lockdowns and social isolation may have amplified certain behaviors related to Internet addiction, particularly social media usage and online gaming. As a result, the findings of this study may reflect a heightened level of digital engagement that could differ from pre-pandemic patterns.

While this study replicated previous research conducted in Italy, we did not specifically explore cross-cultural differences. Although Italy and Spain share some cultural similarities, such as historical, linguistic, and social ties, future studies should explicitly compare findings across different cultural contexts to better understand how cultural factors may influence IA and related behaviors. Cultural factors, such as family dynamics, societal expectations, and media influences, could shape individuals’ perceptions and behaviors regarding technology use.

Future research could benefit from incorporating cross-cultural comparisons, allowing researchers to gain a more nuanced understanding of how cultural and contextual factors interact with individual-level variables, such as personality, coping mechanisms, and social norms, to influence Internet usage patterns and addiction risk.

Additionally, future studies should explore how these behaviors evolve in the post-pandemic context, offering insights into whether the observed trends persist once normal social conditions are restored.

To build upon these findings, longitudinal studies would be particularly valuable in identifying causal relationships between Internet addiction and psychological variables, such as emotional regulation, impulsivity, and personality traits. These studies could track participants over time to determine how specific psychological factors contribute to the development or mitigation of Internet addiction.

Furthermore, examining whether personality traits, such as neuroticism and sensation seeking, moderate the relationships between these variables could provide valuable insights into the dynamics of these behaviors.

Conducting mediation analyses may help clarify the underlying mechanisms that contribute to the observed gender differences. These approaches would enhance the robustness of future research and further illuminate the complex interactions between psychological traits and Internet-related behaviors.

## 6. Conclusions

In recent years, particularly due to the COVID-19 pandemic, the use of digital platforms and the Internet has increased significantly, as these tools facilitated the maintenance of social relationships during challenging times [121]. However, this rise may have also contributed to a higher prevalence of maladaptive behaviors, further impacting mental health. This underscores the urgency of understanding the associated risk factors and exploring any gender differences in the onset of such behaviors.

Our findings support previous literature, revealing distinct patterns of Internet use and addiction susceptibility between males and females. While males exhibit higher engagement in online gaming and risk taking, females demonstrate a stronger inclination towards social media and smartphone usage. These findings highlight the importance of considering gender-specific behaviors and vulnerabilities in both research and intervention strategies. Understanding these differences is crucial for identifying tailored risk and protective factors, which can inform more effective prevention and treatment approaches in the context of digital-era disorders.

In conclusion, the study of gender differences is particularly important for understanding how males and females may experience distinct risk and protective factors related to Internet addiction and other digital-era disorders. These differences can inform the development of gender-specific interventions, both in prevention and treatment, ensuring that approaches are tailored to the unique needs and vulnerabilities of each group. Addressing these differences in future research and clinical practice can contribute to more personalized and effective strategies for mitigating the negative impacts of Internet addiction.

By examining these dynamics in diverse populations, it becomes possible to identify both universal trends and culture-specific behaviors, ultimately facilitating the development of more culturally sensitive prevention and intervention strategies tailored to the unique needs of different communities.

## Figures and Tables

**Table 1:** Comparison of mean scores and standard deviations of “technological” variables according to the participants’ gender.

	Groups	*n*	M	SD	F	*p*	*ή* ^2^
**IAT**	Male	265	39.15	10.97	5.15	0.006	0.017
	Female	320	37.95	9.73			
**BSMAS**	Male	265	13.09	4.96	8.61	0.001	0.029
	Female	320	14.18	4.35			
**SMES**	Male	265	16.06	5.19	6.54	0.002	0.022
	Female	320	16.33	4.47			
**IGDS**	Male	189	16.75	6.13	30.27	0.001	0.089
	Female	125	12.97	4.96			
**PHUB (CD)**	Male	265	10.99	3.70	0.47	0.627	0.002
	Female	320	11.08	3.33			
**PHUB (OBS)**	Male	265	15.21	3.31	8.91	0.001	0.030
	Female	320	16.10	3.07			
**FoMO**	Male	265	23.58	7.04	0.50	0.481	0.001
	Female	320	23.58	7.10			

Note. BSMAS = Bergen Social Media Addiction Scale; SMES = Social Media Engagement Scale; IGDS = Internet Gaming Disorder Scale; PHUB (CD) = phubbing (communication disturbance); PHUB (OBS) = phubbing (phone obsession); FoMO = Fear of Missing Out.

**Table 2:** Comparison of mean scores and standard deviations of personological variables according to the participants’ gender.

	Groups	*n*	M	SD	F	*p*	*ή* ^2^
**DERS**	Male	265	58.69	16.28	9.03	0.001	0.030
	Female	320	60.83	17.09			
**BFI (E)**	Male	265	11.06	2.85	0.28	0.756	0.001
	Female	320	11.20	2.66			
**BFI (N)**	Male	265	7.70	2.79	3.60	0.028	0.012
	Female	320	8.34	2.90			
**BFI (A)**	Male	265	12.58	2.01	3.18	0.042	0.011
	Female	320	12.99	1.92			
**BFI (C)**	Male	265	11.63	2.35	12.573	0.001	0.041
	Female	320	12.51	2.02			
**BFI (O)**	Male	265	10.95	1.62	2.54	0.080	0.009
	Female	320	10.62	1.47			
**PS**	Male	265	61.43	9.49	40.89	0.001	0.066
	Female	320	66.33	9.01			
**UPPS (NU)**	Male	265	10.43	3.79	6.19	0.002	0.021
	Female	320	11.02	4.22			
**UPPS (PU)**	Male	265	11.66	3.41	3.61	0.028	0.012
	Female	320	11.65	3.26			
**UPPS (LPr)**	Male	265	8.83	3.03	1.75	0.174	0.006
	Female	320	8.41	2.87			
**UPPS (LPe)**	Male	265	8.80	3.20	12.63	0.001	0.042
	Female	320	7.56	2.94			
**UPPS (SS)**	Male	265	12.71	3.76	6.48	0.002	0.022
	Female	320	12.07	4.07			

Note. DERS = Difficult in Emotional Regulation Scale; BFI (E) = Big Five Inventory (extraversion); BFI (N) = Big Five Inventory (neuroticism); BFI (A) = Big Five Inventory (agreeableness); BFI (C) = Big Five Inventory (conscientiousness); BFI (O) = Big Five Inventory (openness to experience); PS = Prosociality Scale; UPPS (NU) = Impulsivity Behavior Scale (negative urgency); UPPS (PU) = Impulsivity Behavior Scale (positive urgency); UPPS (LPr) = Impulsivity Behavior Scale (lack of premeditation); UPPS (LPe) = Impulsivity Behavior Scale (lack of perseverance); UPPS (SS) = Impulsivity Behavior Scale (sensation seeking).

**Table 3:** Correlation coefficients (Pearson’s r)—technological variables.

	BMSAS	SMES	IGDS	PHUB (CD)	PHUB (OBS)	FoMO
**Male Group**						
IAT	0.731 **	0.350 **	0.637 **	0.434 **	0.559 **	0.480 **
N	265	265	188	265	265	265
**Female Group**						
IAT	0.728 **	0.399 **	0.298 **	0.499 **	0.566 **	0.450 **
N	320	320	125	320	320	320
**Comparing** **correlations**						
z	0.077	−0.683	3.822 **	−0.996	−0.123	0.459
*p*	0.469	0.247	0.001	0.160	0.451	0.323

Note. ** *p* < 0.001; BSMAS = Bergen Social Media Addiction Scale; SMES = Social Media Engagement Scale; IGDS = Internet Gaming Disorder Scale; PHUB (CD) = phubbing (communication disturbance); PHUB (OBS) = phubbing (phone obsession); FoMO = Fear of Missing Out.

**Table 4:** Correlation coefficients (Pearson’s r)—psychological variables.

	DERS	BFI (E)	BFI (N)	BFI (A)	BFI (C)	BFI (O)	PROS	UPPS (NU)	UPPS (PU)	UPPS (LPr)	UPPS (LPe)	UPPS (SS)
**Male Group**												
IAT	0.454 **	−0.140 **	0.255 **	−0.140 **	−0.094 *	−0.094 *	−0.091	0.278 **	0.182 **	0.130 *	0.173 **	0.059
N	265	265	265	265	265	265	265	265	265	265	265	265
**Female Group**												
IAT	0.428 **	−0.133 **	0.312 **	−0.119 **	−0.178 **	−0.100	−0.091	0.298 **	0.220 **	0.070	0.238 **	0.047
N	320	320	320	320	320	320	320	320	320	320	320	320
**Comparing correlations**												
z	0.387	0.085	−0.743	−0.256	1.026	0.073	0	−0.261	−1.476	0.726	−0.813	0.144
*p*	0.350	0.466	0.229	0.399	0.153	0.471	0.5	0.397	0.07	0.234	0.208	0.443

Note. ** *p* < 0.001, * *p* < 0.01; DERS = Difficult in Emotional Regulation Scale; BFI (E) = Big Five Inventory (extraversion); BFI (N) = Big Five Inventory (neuroticism); BFI (A) = Big Five Inventory (agreeableness); BFI (C) = Big Five Inventory (conscientiousness); BFI (O) = Big Five Inventory (openness to experience); PS = Prosociality Scale; UPPS (NU) = Impulsivity Behavior Scale (negative urgency); UPPS (PU) = Impulsivity Behavior Scale (positive urgency); UPPS (LPr) = Impulsivity Behavior Scale (lack of premeditation); UPPS (LPe) = Impulsivity Behavior Scale (lack of perseverance); UPPS (SS) = Impulsivity Behavior Scale (sensation seeking).

**Table 5:** Multiple linear regression of IAT total score predictors in the male group.

	B	SE B	β	*p*
(Constant)	6.723	2.653		0.012
BMSAS	0.985	0.138	0.436	0.001
IGDS	0.576	0.098	0.319	0.001
PHUB (OBS)	0.578	0.098	0.164	0.002
DERS (AW)	0.406	0.137	0.177	0.004
DERS (NA)	−0.210	0.098	−0.124	0.034
R^2^ = 0.684, F_(5,182)_ = 66.98, *p* < 0.001				

Note. BSMAS = Bergen Social Media Scale; IGDS = Internet Gaming Disorder Scale—Short Form; PHUB (OBS) = phubbing (phone obsession); DERS (AW) = DERS (awareness); DERS (NA) = DERS (non-acceptance).

**Table 6:** Multiple linear regression of IAT total score predictors in the female group.

	B	SE B	β	*p*
(Constant)	19.228	5.022		0.001
BMSAS_TOT	1.301	0.140	0.593	0.001
PHUB (CD)	0.692	0.170	0.255	0.001
IGDS	0.356	0.116	0.182	0.003
PROS	−0.181	0.069	−0.156	0.010
R^2^ = 0.587, F_(4,120)_ = 42.616, *p* < 0.001				

Note. BSMAS = Bergen Social Media Scale; PHUB (CD) = phubbing (communication disturbance); IGDS = Internet Gaming Disorder Scale—Short Form; PROS = prosociality.

## Data Availability

The data are not publicly available because they are part of a larger database, with data that are still being analyzed. The authors are willing to make the data privately available to researchers who request the data.
